# The genome sequence of the swallow prominent, *Pheosia tremula *(Clerck, 1759)

**DOI:** 10.12688/wellcomeopenres.17484.1

**Published:** 2021-12-13

**Authors:** Douglas Boyes, Peter W.H. Holland, University of Oxford and Wytham Woods Genome Acquisition Lab, Darwin Tree of Life Barcoding collective, Wellcome Sanger Institute Tree of Life programme, Wellcome Sanger Institute Scientific Operations: DNA Pipelines collective, Tree of Life Core Informatics collective, Darwin Tree of Life Consortium

**Affiliations:** 1 UK Centre for Ecology and Hydrology, Wallingford, Oxfordshire, UK; 2 Department of ZOology, University of Oxford, Oxford, UK

**Keywords:** Pheosia tremula, swallow prominent, genome sequence, chromosomal, Lepidoptera

## Abstract

We present a genome assembly from an individual male *Pheosia tremula *(the swallow prominent; Arthropoda; Insecta; Lepidoptera; Notodontidae). The genome sequence is 290 megabases in span. The majority of the assembly, 99.94%, is scaffolded into 31 chromosomal pseudomolecules, with the Z sex chromosome assembled.

## Species taxonomy

Eukaryota; Metazoa; Ecdysozoa; Arthropoda; Hexapoda; Insecta; Pterygota; Neoptera; Endopterygota; Lepidoptera; Glossata; Ditrysia; Noctuoidea; Notodontidae; Notodontinae; Pheosia; *Pheosia tremula* (Clerck, 1759) (NCBI:txid988019).

## Background

*Pheosia tremula* (swallow prominent) is a strikingly patterned moth considered ( Allan, 1947) as "aesthetically the perfect insect"; its larvae feed on poplar ( *Populus* sp.) and sallow ( *Salix* sp.). *Pheosia tremula* can be found throughout northern and central Europe and Russia. Within the UK the moth is relatively common in England and Wales but more local in Scotland. The flight period in the UK peaks in May to June, and again in August, and the moth can be found in woodland, plantations, riversides, gardens and parks. Like other moths in the family Notodontidae *, P. tremula* has a single auditory receptor cell associated with each tympanic membrane on the second thoracic segment; *P. tremula* has therefore been used to investigate the electrophysiological basis of auditory reception in simple “one-celled ears” ( Fullard *et al*., 1998). The genome of *P. tremula* was sequenced as part of the Darwin Tree of Life Project, a collaborative effort to sequence all of the named eukaryotic species in the Atlantic Archipelago of Britain and Ireland. Here we present a chromosomally complete genome sequence for *P. tremula*, based on one male specimen from Wytham Woods, Oxfordshire, UK.

## Genome sequence report

**Figure 1.**
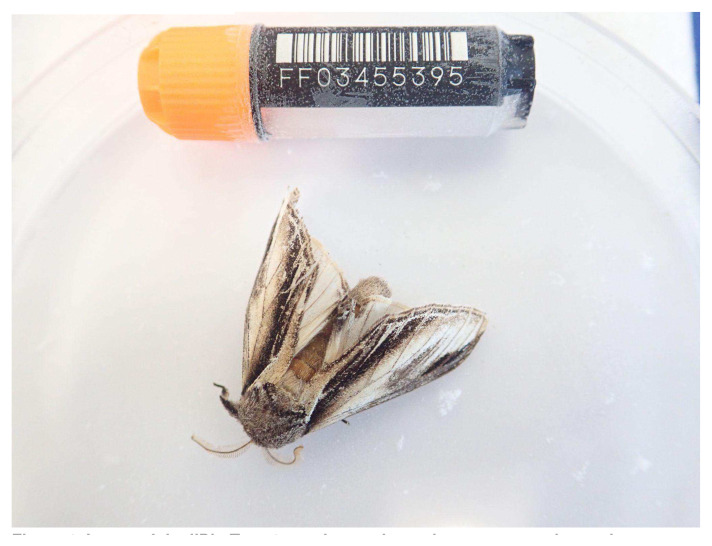
Image of the ilPheTrem1 specimen taken prior to preservation and processing. Specimen shown next to FluidX storage tube, 43.9 mm in length.

The genome was sequenced from a single male *P. tremula* collected from Wytham Woods ( Figure 1), Oxfordshire, UK (latitude 51.768, longitude -1.337). A total of 73-fold coverage in Pacific Biosciences single-molecule long reads and 120-fold coverage in 10X Genomics read clouds were generated. Primary assembly contigs were scaffolded with chromosome conformation Hi-C data. Manual assembly curation corrected 70 missing/misjoins and removed 9 haplotypic duplications, reducing the assembly length by 0.03% and the scaffold number by 48.61%, and increasing the scaffold N50 by 3.55%.

**Figure 2.**
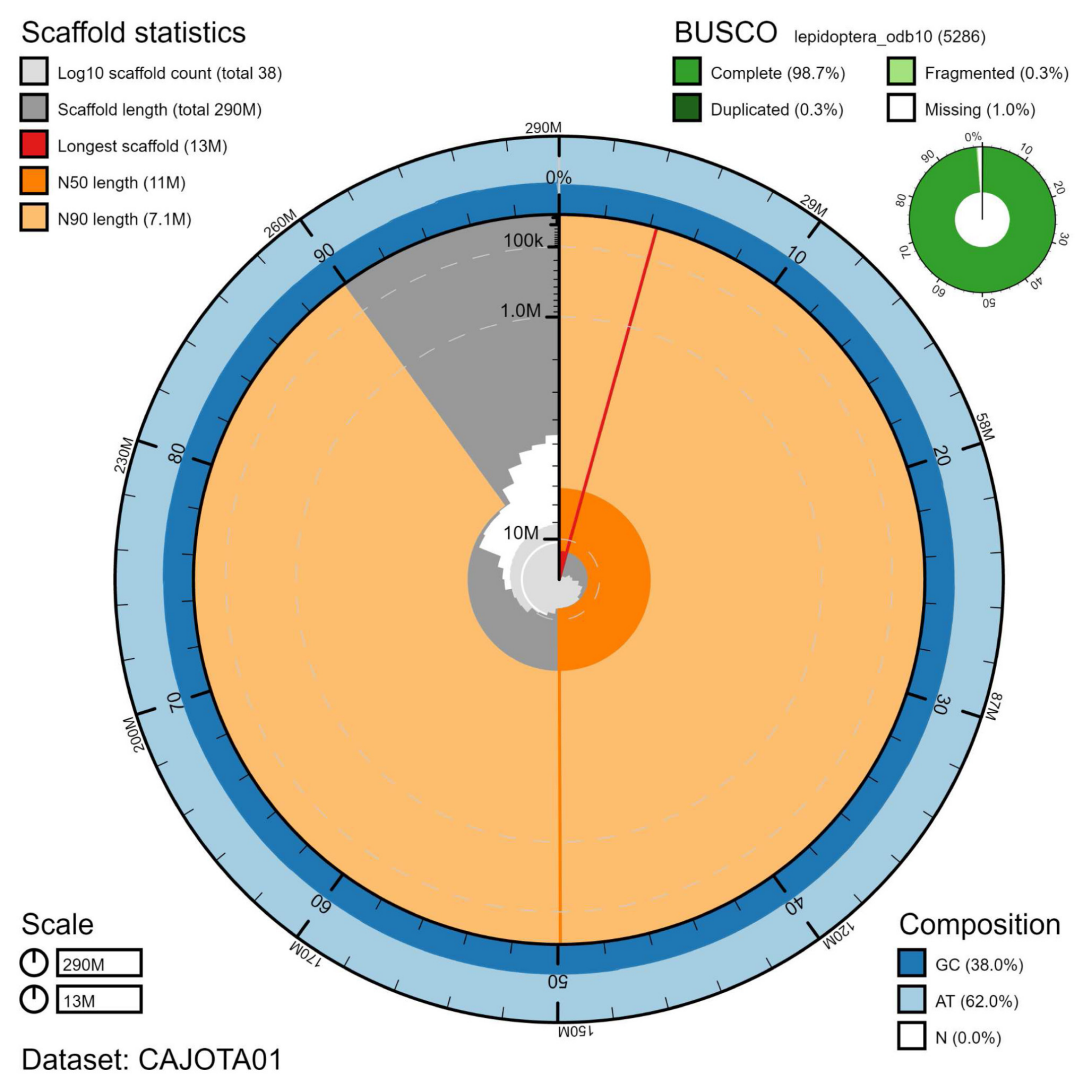
Genome assembly of *Pheosia tremula*, ilPheTrem1.1: metrics. The BlobToolKit Snailplot shows N50 metrics and BUSCO gene completeness. The main plot is divided into 1,000 size-ordered bins around the circumference with each bin representing 0.1% of the 290,221,673 bp assembly. The distribution of chromosome lengths is shown in dark grey with the plot radius scaled to the longest chromosome present in the assembly (12,641,679 bp, shown in red). Orange and pale-orange arcs show the N50 and N90 chromosome lengths (10,632,747 and 7,102,514 bp), respectively. The pale grey spiral shows the cumulative chromosome count on a log scale with white scale lines showing successive orders of magnitude. The blue and pale-blue area around the outside of the plot shows the distribution of GC, AT and N percentages in the same bins as the inner plot. A summary of complete, fragmented, duplicated and missing BUSCO genes in the lepidoptera_odb10 set is shown in the top right. An interactive version of this figure is available at https://blobtoolkit.genomehubs.org/view/ilPheTrem1.1/dataset/CAJOTA01/snail.

The final assembly has a total length of 290 Mb in 38 sequence scaffolds with a scaffold N50 of 11 Mb ( Table 1). Of the assembly sequence, 99.9% was assigned to 31 chromosomal-level scaffolds, representing 30 autosomes (numbered by sequence length), and the Z sex chromosome ( Figure 2– Figure 5; Table 2). The assembly has a BUSCO v5.1.2 ( Manni *et al*., 2021) completeness of 98.7% (single 98.4%, duplicated 0.3%) using the lepidoptera_odb10 reference set. While not fully phased, the assembly deposited is of one haplotype. Contigs corresponding to the second haplotype have also been deposited.

**Table 1.** Genome data for *Pheosia tremula*, ilPheTrem1.1.

*Project accession data*	
Assembly identifier	ilPheTrem1.1
Species	*Pheosia tremula*
Specimen	ilPheTrem1
NCBI taxonomy ID	NCBI:txid988019
BioProject	PRJEB43537
BioSample ID	SAMEA7520523
Isolate information	Male, thorax/abdomen
*Raw data accessions*	
PacificBiosciences SEQUEL II	ERR6412031, ERR6412363
10X Genomics Illumina	ERR6054530-ERR6054533
Hi-C Illumina	ERR6054529
*Genome assembly*	
Assembly accession	GCA_905333125.1
*Accession of alternate haplotype*	GCA_905333115.1
Span (Mb)	290
Number of contigs	106
Contig N50 length (Mb)	7.7
Number of scaffolds	37
Scaffold N50 length (Mb)	10.6
Longest scaffold (Mb)	12.6
BUSCO * genome score	C:98.7%[S:98.4%,D:0.3%], F:0.3%,M:1.0%,n:5286

*BUSCO scores based on the lepidoptera_odb10 BUSCO set using v5.1.2. C= complete [S= single copy, D=duplicated], F=fragmented, M=missing, n=number of orthologues in comparison. A full set of BUSCO scores is available at https://blobtoolkit.genomehubs.org/view/ilPheTrem1.1/dataset/CAJOTA01/busco.

**Figure 3.**
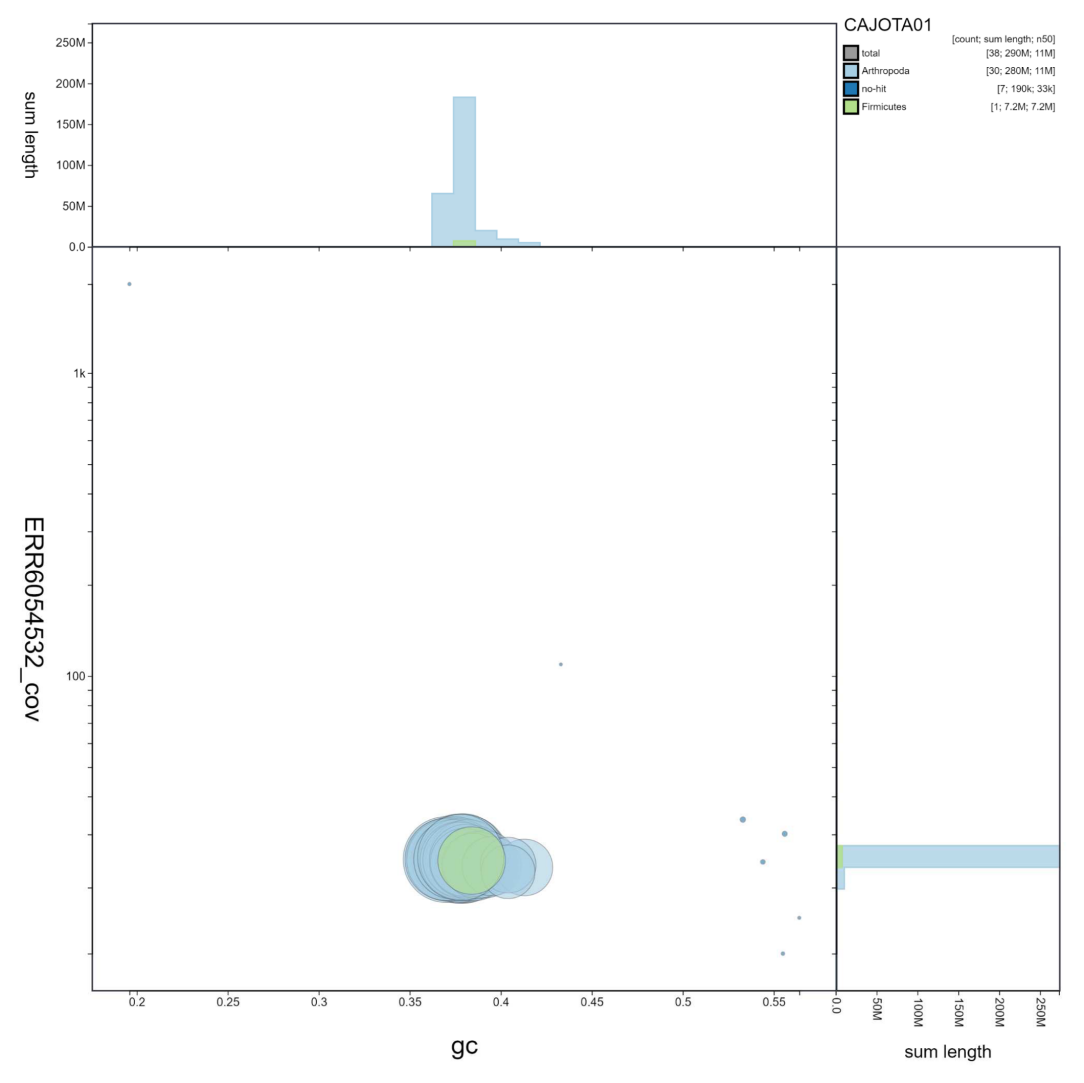
Genome assembly of *Pheosia tremula*, ilPheTrem1.1: GC coverage. BlobToolKit GC-coverage plot. Scaffolds are coloured by phylum. Circles are sized in proportion to scaffold length. Histograms show the distribution of scaffold length sum along each axis. An interactive version of this figure is available at https://blobtoolkit.genomehubs.org/view/ilPheTrem1.1/dataset/CAJOTA01/blob.

**Figure 4.**
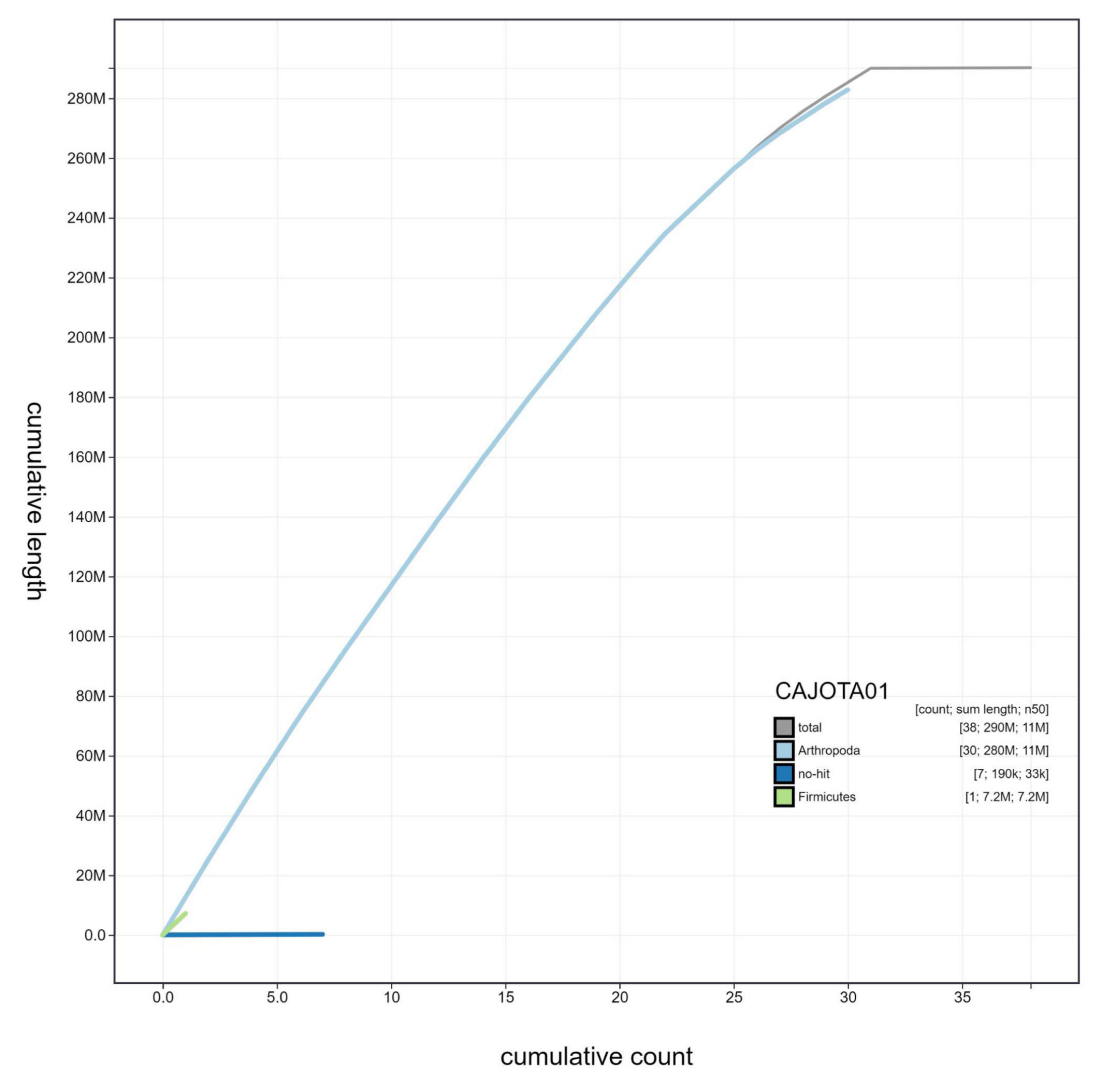
Genome assembly of *Pheosia tremula*, ilPheTrem1.1: cumulative sequence. BlobToolKit cumulative sequence plot. The grey line shows cumulative length for all scaffolds. Coloured lines show cumulative lengths of scaffolds assigned to each phylum using the buscogenes taxrule. An interactive version of this figure is available at https://blobtoolkit.genomehubs.org/view/ilPheTrem1.1/dataset/CAJOTA01/cumulative.

**Figure 5.**
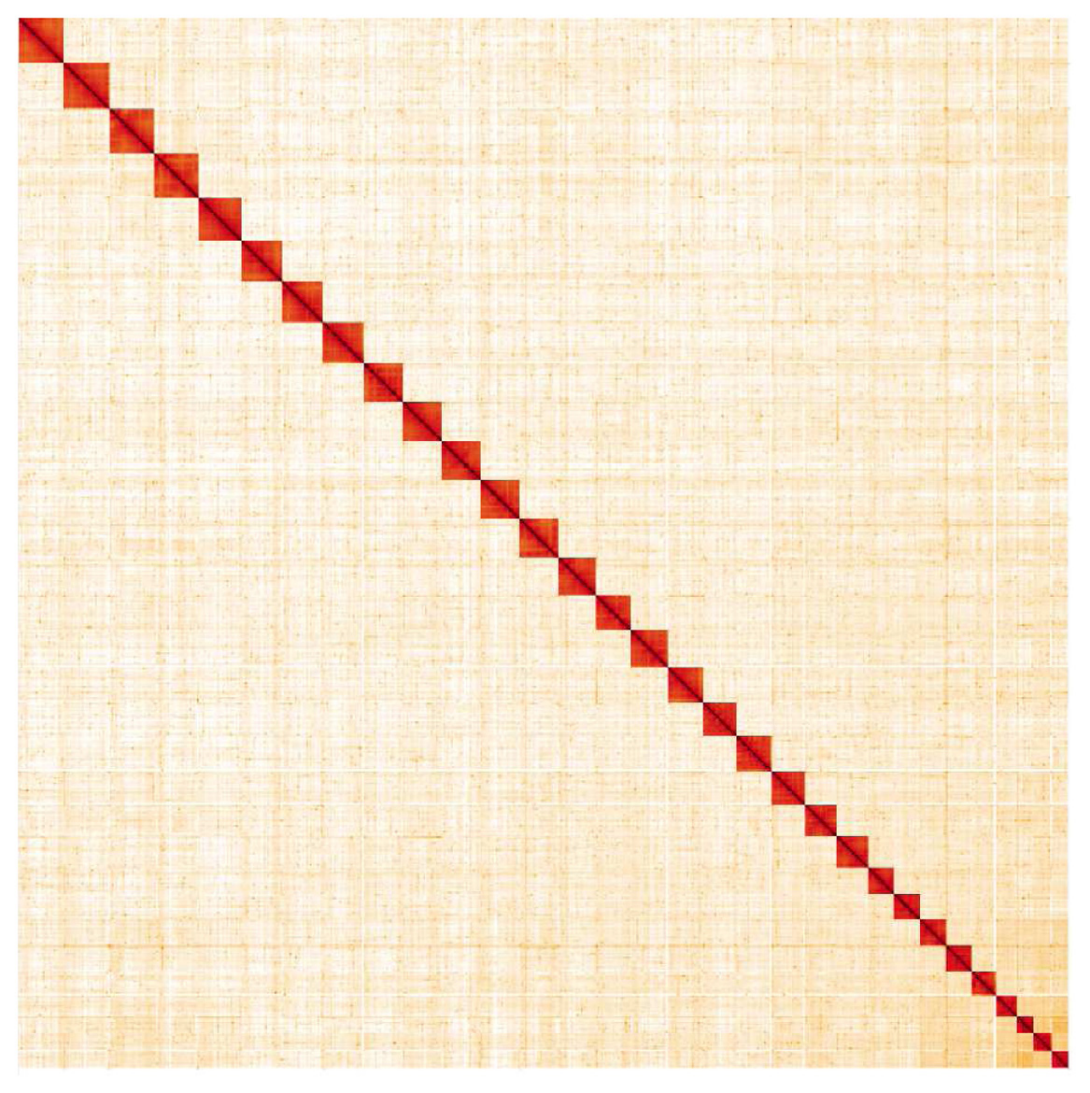
Genome assembly of *Pheosia tremula*, ilPheTrem1.1: Hi-C contact map. Hi-C contact map of the ilPheTrem1.1 assembly, visualised in HiGlass. Chromosomes are given in order of size from left to right and top to bottom.

**Table 2.** Chromosomal pseudomolecules in the genome assembly of *Pheosia tremula*, ilPheTrem1.1.

INSDC accession	Chromosome	Size (Mb)	GC%
HG995397.1	1	12.64	37.9
HG995398.1	2	12.62	37.7
HG995399.1	3	12.25	38
HG995400.1	4	12.19	37.9
HG995402.1	5	11.69	37
HG995403.1	6	11.25	37.4
HG995404.1	7	10.95	37.5
HG995405.1	8	10.89	37.1
HG995406.1	9	10.71	37.7
HG995407.1	10	10.70	37.1
HG995408.1	11	10.69	37
HG995409.1	12	10.63	37.8
HG995410.1	13	10.31	37.7
HG995411.1	14	10.09	37.9
HG995412.1	15	10.01	37.4
HG995413.1	16	9.62	38
HG995414.1	17	9.57	37.7
HG995415.1	18	9.47	38.2
HG995416.1	19	9.08	37.8
HG995417.1	20	8.96	38.5
HG995418.1	21	8.62	38.1
HG995419.1	22	7.33	38
HG995420.1	23	7.21	38.4
HG995421.1	24	7.20	39.1
HG995422.1	25	7.10	38.7
HG995423.1	26	6.30	38.5
HG995424.1	27	5.58	39.5
HG995425.1	28	5.08	41.3
HG995426.1	29	4.91	40.4
HG995427.1	30	4.57	40.4
HG995401.1	Z	11.80	37.8
HG995428.1	MT	0.02	19.5
-	Unplaced	0.17	53.9

## Methods

### Sample acquisition and DNA extraction

A single male *P. tremula* (ilPheTrem1) was collected from Wytham Woods, Oxfordshire, UK (latitude 51.768, longitude -1.337) by Douglas Boyes, UKCEH, using a light trap. The specimen was identified by the same individual and preserved on dry ice.

DNA was extracted at the Tree of Life laboratory, Wellcome Sanger Institute. The ilPheTrem1 sample was weighed and dissected on dry ice with tissue set aside for Hi-C sequencing. Thorax/abdomen tissue was cryogenically disrupted to a fine powder using a Covaris cryoPREP Automated Dry Pulveriser, receiving multiple impacts. Fragment size analysis of 0.01–0.5 ng of DNA was then performed using an Agilent FemtoPulse. High molecular weight (HMW) DNA was extracted using the Qiagen MagAttract HMW DNA extraction kit. Low molecular weight DNA was removed from a 200-ng aliquot of extracted DNA using 0.8X AMpure XP purification kit prior to 10X Chromium sequencing; a minimum of 50 ng DNA was submitted for 10X sequencing. HMW DNA was sheared into an average fragment size between 12–20 kb in a Megaruptor 3 system with speed setting 30. Sheared DNA was purified by solid-phase reversible immobilisation using AMPure PB beads with a 1.8X ratio of beads to sample to remove the shorter fragments and concentrate the DNA sample. The concentration of the sheared and purified DNA was assessed using a Nanodrop spectrophotometer and Qubit Fluorometer and Qubit dsDNA High Sensitivity Assay kit. Fragment size distribution was evaluated by running the sample on the FemtoPulse system.

### Sequencing

Pacific Biosciences HiFi circular consensus and 10X Genomics read cloud sequencing libraries were constructed according to the manufacturers’ instructions. Sequencing was performed by the Scientific Operations core at the Wellcome Sanger Institute on Pacific Biosciences SEQUEL II and Illumina HiSeq X instruments. Hi-C data were generated from thorax/abdomen tissue using the Arima Hi-C+ kit and sequenced on HiSeq X.

### Genome assembly

Assembly was carried out with HiCanu ( Nurk *et al*., 2020). Haplotypic duplication was identified and removed with purge_dups ( Guan *et al*., 2020). Scaffolding with Hi-C data ( Rao *et al*., 2014) was carried out with SALSA2 ( Ghurye *et al*., 2019). The Hi-C scaffolded assembly was polished with the 10X Genomics Illumina data by aligning to the assembly with longranger align, calling variants with freebayes ( Garrison & Marth, 2012). One round of the Illumina polishing was applied. The mitochondrial genome was assembled with MitoHiFi ( Uliano-Silva *et al*., 2021) and annotated using MitoFinder ( Allio *et al*., 2020). The assembly was checked for contamination and corrected using the gEVAL system ( Chow *et al*., 2016) as described previously ( Howe *et al*., 2021). Manual curation ( Howe *et al*., 2021) was performed using gEVAL, HiGlass ( Kerpedjiev *et al*., 2018) and Pretext. The genome was analysed within the BlobToolKit environment ( Challis *et al*., 2020). Table 3 contains a list of all software tool versions used, where appropriate.

**Table 3.** Software tools used.

Software tool	Version	Source
HiCanu	2.1	Nurk *et al*., 2020
purge_dups	1.2.3	Guan *et al*., 2020
SALSA2	2.2	Ghurye *et al*., 2019
longranger align	2.2.2	https://support.10xgenomics.com/genome-exome/ software/pipelines/latest/advanced/other-pipelines
freebayes	v1.3.1-17-gaa2ace8	Garrison & Marth, 2012
MitoHiFi	1.0	Uliano-Silva *et al*., 2021
gEVAL	N/A	Chow *et al*., 2016
HiGlass	1.11.6	Kerpedjiev *et al*., 2018
PretextView	0.1.x	https://github.com/wtsi-hpag/PretextView
BlobToolKit	2.6.2	Challis *et al*., 2020

### Ethics/compliance issues

The materials that have contributed to this genome note have been supplied by a Darwin Tree of Life Partner. The submission of materials by a Darwin Tree of Life Partner is subject to the Darwin Tree of Life Project Sampling Code of Practice. By agreeing with and signing up to the Sampling Code of Practice, the Darwin Tree of Life Partner agrees they will meet the legal and ethical requirements and standards set out within this document in respect of all samples acquired for, and supplied to, the Darwin Tree of Life Project. Each transfer of samples is further undertaken according to a Research Collaboration Agreement or Material Transfer Agreement entered into by the Darwin Tree of Life Partner, Genome Research Limited (operating as the Wellcome Sanger Institute), and in some circumstances other Darwin Tree of Life collaborators.

## Data availability

European Nucleotide Archive: Pheosia tremula (swallow prominent). Accession number PRJEB43537; https://www.ebi.ac.uk/ena/browser/view/PRJEB43537.

The genome sequence is released openly for reuse. The *P. tremula* genome sequencing initiative is part of the Darwin Tree of Life (DToL) project. All raw sequence data and the assembly have been deposited in INSDC databases. The genome will be annotated and presented through the Ensembl pipeline at the European Bioinformatics Institute. Raw data and assembly accession identifiers are reported in Table 1.

## Author information

Members of the University of Oxford and Wytham Woods Genome Acquisition Lab are listed here: https://doi.org/10.5281/zenodo.5746938.

Members of the Darwin Tree of Life Barcoding collective are listed here: https://doi.org/10.5281/zenodo.5744972.

Members of the Wellcome Sanger Institute Tree of Life programme are listed here: https://doi.org/10.5281/zenodo.5744840.

Members of Wellcome Sanger Institute Scientific Operations: DNA Pipelines collective are listed here: https://doi.org/10.5281/zenodo.5746904.

Members of the Tree of Life Core Informatics collective are listed here: https://doi.org/10.5281/zenodo.5743293.

Members of the Darwin Tree of Life Consortium are listed here: https://doi.org/10.5281/zenodo.5638618.

## References

[ref-1] Allan PBM : A Moth Hunter’s Gossip (Watkins & Doncaster). Moths and Memories (Watkins & Doncaster).1947. Reference Source

[ref-2] Allio R Schomaker-Bastos A Romiguier J : MitoFinder: Efficient Automated Large-Scale Extraction of Mitogenomic Data in Target Enrichment Phylogenomics. *Mol Ecol Resour.* 2020;20(4):892–905. 10.1111/1755-0998.13160 32243090 PMC7497042

[ref-3] Challis R Richards E Rajan J : BlobToolKit--Interactive Quality Assessment of Genome Assemblies. *G3 (Bethesda).* 2020;10(4):1361–1374. 10.1534/g3.119.400908 32071071 PMC7144090

[ref-4] Chow W Brugger K Caccamo M : gEVAL — a Web-Based Browser for Evaluating Genome Assemblies. *Bioinformatics.* 2016;32(16):2508–10. 10.1093/bioinformatics/btw159 27153597 PMC4978925

[ref-5] Fullard JH Forrest E Surlykke A : Intensity Responses of the Single Auditory Receptor of Notodontid Moths: A Test of the Peripheral Interaction Hypothesis in Moth Ears. *J Exp Biol.* 1998;201(Pt 24):3419–24. 9817838 10.1242/jeb.201.24.3419

[ref-6] Garrison E Marth G : Haplotype-Based Variant Detection from Short-Read Sequencing, arXiv: 1207.3907.2012. Reference Source

[ref-7] Ghurye J Rhie A Walenz BP : Integrating Hi-C Links with Assembly Graphs for Chromosome-Scale Assembly. *PLoS Comput Biol.* 2019;15(8):e1007273. 10.1371/journal.pcbi.1007273 31433799 PMC6719893

[ref-8] Guan D McCarthy SA Wood J : Identifying and Removing Haplotypic Duplication in Primary Genome Assemblies. *Bioinformatics.* 2020;36(9):2896–2898. 10.1093/bioinformatics/btaa025 31971576 PMC7203741

[ref-9] Howe K Chow W Collins J : Significantly Improving the Quality of Genome Assemblies through Curation. *Gigascience.* 2021;10(1):giaa153. 10.1093/gigascience/giaa153 33420778 PMC7794651

[ref-10] Kerpedjiev P Abdennur N Lekschas F : HiGlass: Web-Based Visual Exploration and Analysis of Genome Interaction Maps. *Genome Biol.* 2018;19(1):125. 10.1186/s13059-018-1486-1 30143029 PMC6109259

[ref-11] Manni M Berkeley MR Seppey M : BUSCO Update: Novel and Streamlined Workflows along with Broader and Deeper Phylogenetic Coverage for Scoring of Eukaryotic, Prokaryotic, and Viral Genomes. *Mol Biol Evol.* 2021;38(10):4647–54. 10.1093/molbev/msab199 34320186 PMC8476166

[ref-12] Nurk S Walenz BP Rhie A : HiCanu: Accurate Assembly of Segmental Duplications, Satellites, and Allelic Variants from High-Fidelity Long Reads. *Genome Res.* 2020;30(9):1291–1305. 10.1101/gr.263566.120 32801147 PMC7545148

[ref-13] Rao SSP Huntley MH Durand NC : A 3D Map of the Human Genome at Kilobase Resolution Reveals Principles of Chromatin Looping. *Cell.* 2014;159(7):1665–80. 10.1016/j.cell.2014.11.021 25497547 PMC5635824

[ref-14] Uliano-Silva M Nunes JGF Krasheninnikova K : marcelauliano/MitoHiFi: mitohifi_v2.0.2021. 10.5281/zenodo.5205678

