# Supplementary material for: The genome sequence of the swallow prominent, Pheosia tremula (Clerck, 1759)
Source: Wellcome Open Res. 2021 Dec 13;6:335. [Version 1] doi: 10.12688/wellcomeopenres.17484.1 (PMC9975399; doi:10.12688/wellcomeopenres.17484.1)
Supplement: Peer review history

# Peer review: Reviewer response for version 1

**DOI:** 10.21956/wellcomeopenres.19334.r53442

**Published:** 2022-12-08

**Authors:** Yuttapong Thawornwattana

**Affiliations:** 1 Department of Organismic and Evolutionary Biology, Harvard University, Cambridge, USA

Boyes & Holland report a chromosome-level genome assembly of *Pheosia tremula* (Clerck, 1759) (Lepidoptera: Notodontidae) based on high-coverage PacBio HiFi long reads from a single male individual. The assembly was scaffolded with Hi-C data and polished with 10X Genomics Illumina reads, produced as part of the Darwin Tree of Life project.  

 The assembly is of high-quality, with both scaffold N50 and N90 in a megabase range, and high BUSCO assembly completeness (>98% for single-copy orthologs in Lepidoptera). However, genome annotation was not mentioned. The status of the W chromosome was also not mentioned. There was notable a presence of firmicutes bacteria from taxonomic annotation of scaffolds could be due to contamination or symbiosis.

Are sufficient details of methods and materials provided to allow replication by others?

Yes

Is the rationale for creating the dataset(s) clearly described?

Yes

Are the datasets clearly presented in a useable and accessible format?

Yes

Are the protocols appropriate and is the work technically sound?

Yes

Reviewer Expertise:

phylogenetics, evolutionary genomics

I confirm that I have read this submission and believe that I have an appropriate level of expertise to confirm that it is of an acceptable scientific standard.

# Peer review: Reviewer response for version 1

**DOI:** 10.21956/wellcomeopenres.19334.r47665

**Published:** 2022-01-19

**Authors:** Simon H. Martin

**Affiliations:** 1 Institute of Evolutionary Biology, University of Edinburgh, Edinburgh, UK

The authors present a highly contiguous and complete haploid genome assembly for a male Pheosia tremula. This excellent assembly would be of value as a reference genome and for comparative genomics projects.

 I have only a few minor requests for improved clarity:

1. Please specify the read length for the Illumina sequencing.
2. Prior to scaffolding with SLASA2, I believe it is necessary to align the HI-C reads to the contigs. As far as I could tell, the alignment approach is not described.
3. The large contig that is labelled as Firmicutes in Figures 3 and 4 is not mentioned in the main text. Was this interpreted as contamination and excluded from the final assembly?

Are sufficient details of methods and materials provided to allow replication by others?

Partly

Is the rationale for creating the dataset(s) clearly described?

Yes

Are the datasets clearly presented in a useable and accessible format?

Yes

Are the protocols appropriate and is the work technically sound?

Yes

Reviewer Expertise:

Evolution, genomics and population genetics

I confirm that I have read this submission and believe that I have an appropriate level of expertise to confirm that it is of an acceptable scientific standard.
